# Notes and News

**Published:** 1906-03-03

**Authors:** 


### Notes and News.

On Tuesday afternoon the Medical Members' Committee at the House of Commons met and selected Sir Walter Foster as Chairman. A number of questions were discussed, including vaccination, vivisection, and the position of medical referees under the Compensation Act.

The Foleshill Rural District Council in Warwickshire last year sent two cases of small-pox from their workhouse to the County Isolation Hospital, for which they have just had to pay £71 each. Three years ago they treated 36 cases in their own hospital at a cost per patient of £14 11s.

At a meeting in Leeds last Friday, which was attended by several medical officers of health, the Mayor of Huddersfield moved that a national conference to consider the question of infantile mortality should be held in London. No decision was arrived at in respect to the date, but the month of June was mentioned.

A Provision merchant at Birmingham has been fined £20 and £30 costs for supplying to the City Hospital margarine instead of butter which he had contracted to supply. The margarine was placed in Danish butter casks, and it was proved that these were obtained empty from a dealer who received butter from Denmark. The difference in the price of the two articles was 50s. per cwt.

In order to facilitate the policy of concentration and the complete separation of the department of preliminary and intermediate medical studies carried on at King's College from the department of advanced medical studies carried on at King's College Hospital, Professor F. W. Tunnicliffe, M.D., who holds appointments in both departments, has resigned the Chair of Materia Medica and Pharmacology.

At the annual meeting of the Royal Isle of Wight County Hospital, held on Monday, it was decided to proceed at once with the second part of the hospital improvement scheme, comprising provision of a new out-patients' department and isolation wards, at a cost of about £5,000.

This week the line from Neasden to Akeman Street, passing through South Harrow, Beaconsfield, High Wycombe, and Princes Risboro', is opened for passenger traffic. The new suburban route, whose terminus is, of course, Marylebone, will offer attractions to many members of the medical profession in London, especially as the travelling facilities offered by the Great Central Railway Company are exceptionally good.

Dr. Claude St. Aubyn-Farrer presided at the annual dinner of the Association of Medical Diplomates of Scotland in London last week, and stated that the association has already done a good deal in the way of placing its views before the councils of the various Scottish corporations, much to the advantage of its members. The toast of the evening was proposed by Dr. C. W. MacGillivray, who assured the company that he and his colleagues were doing their very best to promote their welfare.

The twenty-third annual Congress of the Royal Sanitary Institute will be held at Bristol from July 9 to 14. The Right Hon. Sir Edward Fry has consented to act as President of the Congress and the Duchess of Beaufort will preside over the Ladies' Conference. The Congress will be divided into three sections, as follows: Section I., Sanitary Science and Preventive Medicine; Section II., Engineering and Architecture; Section III., Physics, Chemistry, and Biology. The presidents of these sections will be, respectively, Sir William J. Collins, Mr. Edwin T. Hall, and Dr. W. N. Shaw.

In presenting his report at the annual meeting of the corporation of the Royal Edinburgh Asylum for the Insane, which was held on Monday, Dr. Clouston called attention to a remarkable fact—namely, the large increase of general paralysis, especially among women. He could remember the time when a case of general paralysis in a woman was so uncommon that on the admission of such a case all the medical staff would go to examine the patient. Indeed, sometimes a whole year would pass without the admission of a single case of this kind. In contrast with this, no fewer than 38 women suffering from the disease had been admitted in the course of last year. As a matter of fact, the number of female general paralytics admitted during that period exceeded that of the men; a phenomenon which was unprecedented. Dr. Clouston referred to the investigations which have recently been carried out by Drs. Ford Robertson and M'Rae as to the cause of the disease, and expressed himself as a believer in the microbic hypothesis which they have propounded. The cheering part of their investigation, he thought, was that it pointed to the possible discovery in the future of a cure for general paralysis.

